# Feedback Intervention Using a Wearable Activity Monitor in a Patient With Early-Stage Upper Limb Complex Regional Pain Syndrome: A Case Report

**DOI:** 10.7759/cureus.115691

**Published:** 2026-09-02

**Authors:** Kazuyo Tsukamoto, Yuki Hiraga, Takamichi Taniguchi

**Affiliations:** 1 Department of Occupational Therapy, International University of Health and Welfare Graduate School, Fukuoka, JPN; 2 Department of Occupational Therapy, Faculty of Medicine, Fukuoka International University of Health and Welfare, Fukuoka, JPN; 3 Department of Occupational Therapy, School of Health Sciences, International University of Health and Welfare Graduate School, Otawara, JPN

**Keywords:** activity pacing, crps, japanese rehabilitation, rehabilitation, self-regulation, wearable devices

## Abstract

Patients in the early stages of Complex Regional Pain Syndrome (CRPS) of the upper extremities are unable to accurately perceive their own activity levels because of fear-avoidance beliefs and distortions in body perception, making them prone to falling into a “boom-and-bust cycle” characterized by alternating periods of overactivity and inactivity. The purpose of this case report is to examine the effect of a feedback intervention using an activity tracker (Fitbit) on “self-regulatory behavior.” A woman in her 50s who worked as both an interpreter and translator was diagnosed with upper limb CRPS because of persistent pain following right-hand surgery. Due to her strong sense of responsibility and perfectionist personality, she exhibited a maladaptive activity pattern in which she forced herself to use her right hand even under severe pain, only to cease all activity for several days once she reached her limit. Initial assessments showed a Disabilities of the Arm, Shoulder, and Hand (DASH) score of 78, a Pain Catastrophizing Scale (PCS) score of 35, a Tampa Scale for Kinesiophobia score of 48, and a Pain Self-Efficacy Questionnaire (PSEQ) score of 23. The Fitbit data revealed exceptionally high activity (52 minutes) on a specific day, which the patient was unaware of.

The intervention centered on “self-regulation” to correct distorted body perception based on objective data, and the following step-by-step program was implemented over eight weeks. (i) Initial Phase: feedback was provided through a three-step process - presentation of Fitbit data, verification of the patient’s perception, and development of a specific action plan - and “Activity Pacing” was taught while correcting recall bias. (ii) Middle Phase: once resting pain was reduced through self-regulation, sensory re-education was conducted to correct pain predictions using a desensitization protocol with graded materials, body regions, and modalities. (iii) Final Phase: we applied gradual functional loading based on the morphological characteristics of objects and integrated the strategic self-regulation of “activity selection and concentration” into the performance of real-life roles. The DASH score improved from 78 to 57 (a 21-point improvement), and the PCS score improved from 35 to 26 (a nine-point improvement); both results exceeded the minimum clinically important difference (MCID). The Numerical Rating Scale (NRS) score at rest decreased from 10 to 3. Qualitatively, the patient acquired the ability to use the Fitbit data as a “mirror for self-regulation,” and adaptive self-management behaviors - prioritizing energy allocation for important tasks while managing pain - were developed. Quantitative feedback from an activity tracker may be a useful intervention tool for promoting functional recovery and active behavioral changes in patients with early-stage CRPS by enabling them to self-regulate their subjective perception of activity based on actual physiological responses and by curbing disorganized overactivity.

## Introduction

In persistent postsurgical pain, a patient's subjective perception of functional impairment often acts as a critical predictor of subsequent pain intensity [1,2]. Our previous study demonstrated that patient-reported subjective disability, rather than objective physical functions such as grip strength, was the strongest predictor of six-month postoperative pain following lateral epicondylitis (LE) surgery [3]. The clinical importance of subjective experiences becomes even more vital in Complex Regional Pain Syndrome (CRPS). Typically developing after trauma or surgery, upper-extremity CRPS is characterized by disproportionate localized pain, autonomic dysfunction, and profound distortions in body perception. In CRPS, a patient’s subjective perception of activity often diverges from their actual activity level and physiological responses, driven by the fear of pain and a tendency to excessively protect the affected limb - commonly conceptualized as fear-avoidance beliefs [2,3]. Specifically, inactivity, extreme avoidance of the affected limb (learned non-use), and overactivity, engaging in activity beyond one’s tolerance and resulting in worsened pain or severe fatigue, may coexist, forming a “boom-and-bust” cycle [2,4]. In patients with early-stage CRPS, it is particularly difficult to reconcile the discrepancy between subjective perceptions of activity and objective activity status. Consequently, pacing may be difficult [5]. Conventional, point-in-time clinical evaluations conducted in a hospital setting typically measure a patient's “capacity” (what they can do in a structured environment). However, such assessments fail to capture real-life “performance” (what they actually do in daily life) and the highly fluctuating overactivity that patients themselves often fail to recognize [6,7].

Recently, the quantification of activity levels using wearable devices has enabled the visualization of the gap between patients’ subjective perceptions of activity and their objective activity levels [6,7]. Furthermore, by providing patients with feedback on objective activity data, they become less susceptible to recall bias and can reassess their subjective perception of activity in light of actual activity data [5,7]. However, there are few reports applying such objective activity feedback to Activity Pacing to support self-regulation of activity in patients with early-stage CRPS who exhibit a “boom-and-bust” cycle. Here, the “boom-and-bust” cycle represents a maladaptive activity pattern, whereas Activity Pacing is a behavioral strategy to interrupt this cycle, and self-regulation is the process of using objective activity feedback to adjust activity accordingly.

Previously, we examined the predictors of pain intensity six months post-LE surgery and found that, compared with objective measures of physical function such as grip strength and range of motion, the subjective experience of functional impairment felt by patients in daily life, as assessed by the Japanese version of the Patient-Rated Tennis Elbow Evaluation (PREE-J), was the strongest predictor of pain intensity [8]. This suggests that, to understand persistent pain following LE surgery, it is necessary to consider both the objective aspects, such as physical function and activity level, and the subjective aspect of how patients perceive their upper limb function [8]. Therefore, in this case report, we focused on the discrepancy between the patient’s subjective perception of activity and their objective activity level and considered that visualizing this discrepancy through feedback on objective activity levels may support the self-regulation of activity. In this study, a Fitbit device was specifically selected because of its lightweight, non-invasive design and high user accessibility, which allowed the patient to easily monitor “active minutes” in real time. This immediate visual accessibility serves as a practical, real-time “mirror” to correct the patient's recall bias during daily activities and facilitate independent self-regulation.

The purpose of this case report was to use an activity tracker (Fitbit) to provide feedback on objective activity levels and visualize the discrepancy between subjective perceptions of activity and objective activity levels in a patient with early-stage CRPS exhibiting a “boom-and-bust” cycle. Through this, we aimed to examine the preliminary effects of a process designed to encourage self-regulation of activity toward correcting maladaptive activity patterns and discuss its clinical significance.

## Case presentation

The patient was a right-handed woman in her 50s. She worked as both an interpreter and translator. Her daily work involved taking rapid notes and typing for long periods, which required a high degree of manual dexterity and precision. She had a strong sense of responsibility and exhibited perfectionist tendencies. At home, she felt a strong sense of responsibility as a homemaker and held a sense of duty, believing that “I must perform household chores perfectly” and “I should be the one to care for my parents.”

Regarding the numbness in her right hand, she had been diagnosed with De Quervain’s tenosynovitis by a previous physician and had undergone six months of conservative treatment. However, her symptoms did not improve and worsened to severe pain in the pads of her thumb and index finger. This led to her referral to our hospital, where she was diagnosed with a combination of carpal tunnel syndrome and De Quervain’s tenosynovitis. On day 0, right-hand carpal tunnel release and tenosynovectomy were performed. A preoperative anteroposterior X-ray image of the right hand, obtained during the initial orthopedic consultation prior to the surgery, is shown in Figure 1. Within two weeks postoperatively (around postoperative day (POD) 14), severe pain, swelling, and limited movement of her right thumb rapidly progressed, indicating the onset of CRPS symptoms. She continued physical therapy at the referring clinic under the guidance of a physician; however, her pain did not subside. One month postoperatively, she was prescribed medications, including celecoxib, Neurotropin, and Talijé, but adequate pain control was not achieved. Two months postoperatively (72 days post-surgery, which marked her baseline assessment), she presented with severe pain, a sensation of finger tightness, and significant difficulty in using chopsticks and subsequently began rehabilitation. At this point, her total disease duration was approximately eight months from the initial onset of right-hand numbness and pain. At the initial evaluation, even a slight touch across the entire dorsum of the hand and around the surgical incision on the palmar, radial side of the wrist was painful. Allodynia, accompanied by a marked flexion-avoidance response, was also observed. After consulting the Department of Anesthesiology, the patient was diagnosed with early-stage upper-extremity CRPS strictly in accordance with the Budapest clinical diagnostic criteria. At this point, the patient held the belief that “food doesn’t taste good unless I eat with my right hand, which is my dominant hand” and “I can’t work without using my right hand,” demonstrating a strong fixation on using her right hand and a sense of impatience. Consequently, she repeatedly followed a pattern of continuing to use her right hand despite the pain, switching to her left hand only when pain or fatigue made it difficult to continue using her right hand. This deeply ingrained pattern had persisted since her surgery; indeed, on the day of surgery, right-hand carpal tunnel release and tenosynovectomy were performed. A radiograph (anterior view) is shown in Figure 1. Throughout this clinical course, this activity pattern created a disconnect between the patient’s desired role performance and the activity adjustments necessary to protect the affected limb and manage pain.

**Figure 1:**
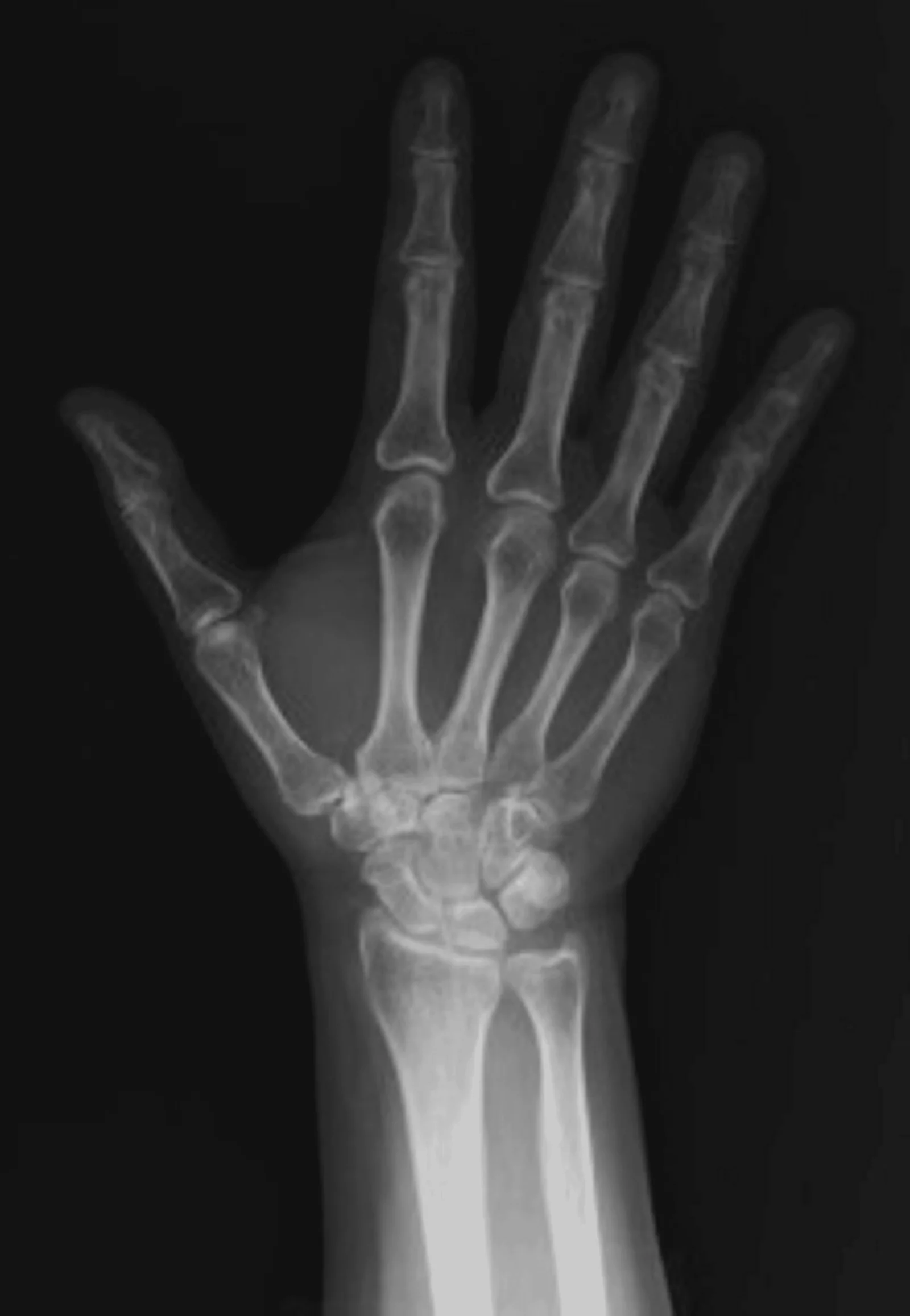
Preoperative Anteroposterior Radiograph of the Right Hand The radiograph image confirms the skeletal status of the right hand prior to the carpal tunnel release and tenosynovectomy performed on the operated side.

Clinical findings

The initial occupational therapy assessment was conducted 72 days post-surgery. Evaluation began with semi-structured interviews based on the Canadian Occupational Performance Measure (COPM) [9] to identify occupational activities of high importance. In this case, “being able to eat using chopsticks” was identified as the highest priority, with both initial performance and satisfaction scores of 1.0. In addition to the subjective functional assessment, joint range of motion was measured using a universal goniometer according to the standards of the American Academy of Orthopaedic Surgeons [10]. Measurements were performed in a seated position, with the upper arm stabilized against the trunk and the radial shaft serving as the reference axis. The results showed that the active range of motion (AROM) for wrist palmar flexion (right/left) was 50°/70°, and dorsiflexion was 35°/70°. Furthermore, concerning the thumb, interphalangeal (IP) joint flexion was 80°/80°, and extension was -10°/-10°; metacarpophalangeal (MP) joint flexion was 30°/0°, and extension was 0°/10°. However, owing to marked allodynia, passive range of motion measurements involving physical contact with the examiner could not be performed.

Subsequently, sensory function was evaluated, while care was taken to avoid the application of pressure stimuli during the assessment. The examiner instructed the patient to say “yes” when each fingertip was touched, and the test was performed five times for each finger. The results indicated that superficial sensations were rated 5/5 for all fingers of the right hand; however, upon contact with the thumb and index fingers, the patient experienced “considerable pain.” Regarding the unaffected side, the same test was performed, and a 5/5 response was observed in all fingers.

To evaluate pain intensity and psychological status, the Numerical Rating Scale (NRS) [11] was utilized, where 0 represents no pain and 10 represents the worst imaginable pain. At the baseline assessment, the patient was asked to rate her current pain intensity, and both pain at rest and pain during movement were rated as 10/10 on the NRS. Although having equal resting and movement pain scores is clinically uncommon, this presentation was considered to reflect the patient’s severe hypersensitivity and distorted body perception, wherein even the slightest ambient stimulation or resting state triggered maximum subjective pain.

Additionally, catastrophic thinking regarding pain was assessed using the Pain Catastrophizing Scale (PCS) [12], which comprises three subscales: rumination, helplessness, and magnification. Higher scores indicate a stronger tendency toward catastrophic thinking; in this case, the patient’s scores were 19/20 for rumination, 9/20 for helplessness, and 7/12 for magnification, totaling 35 out of 52. Furthermore, fear-avoidance thinking - involving excessive restriction of movement due to anxiety - was measured with the Tampa Scale for Kinesiophobia (TSK) [2]. The patient’s score of 48 out of 68 points exceeded the established cutoff value of 37.

Moreover, pain self-efficacy was evaluated using the Pain Self-Efficacy Questionnaire (PSEQ) [13]. This 10-item scale ranges from 0 to 6 per item, with lower scores indicating lower confidence in performing activities while in pain; here, the patient scored 23 out of 60, falling below the cutoff of 40 points. Finally, the Shortened Central Sensitization Inventory (CSI-9) [14] was employed as a screening tool for central sensitization syndrome. Although her score of 18 out of 36 did not reach the cutoff of 20, accompanying symptoms such as insomnia and generalized pain suggested the involvement of central sensitization.

Upper limb functional impairment was assessed using the Disabilities of the Arm, Shoulder, and Hand (DASH) questionnaire [15]. The DASH questionnaire consists of 30 items related to upper limb functional impairment and symptoms, with higher scores indicating more severe functional impairment; in this case, the patient’s initial score was 78 out of 100. Regarding her activities of daily living (ADL) and instrumental activities of daily living (IADL), the patient consistently used her operated right upper limb to manipulate chopsticks and hold a pen. Specifically, when these objects contacted the radial-dorsal side of the index finger’s proximal phalanx shaft or the transition zone between the thumb and index finger, she reported pain far exceeding 10 on the NRS.

Despite this severe pain, the patient maintained a strong preference for using her dominant hand, stating, “I can’t taste my food or write unless I use my right hand.” Consequently, she repeatedly used her right hand until her pain tolerance was reached before switching to her left hand. Furthermore, this tendency toward overactivity was exacerbated by a sense of urgency to return to work as an interpreter and a perfectionist belief that she must perform housework perfectly without relying on others. Given this background, the patient demanded a high level of performance in both daily and occupational activities, which likely contributed to the repetitive overuse of her right thumb and index finger and the subsequent worsening of her pain. These comprehensive functional and psychological assessment results at baseline are summarized in Table 1.

**Table 1:** Summary of Functional and Psychological Evaluation Results for the Patient COPM: Canadian Occupational Performance Measure; ROM: Range of Motion; DASH: Disabilities of the Arm, Shoulder, and Hand; NRS: Numerical Rating Scale; PCS: Pain Catastrophizing Scale; TSK: Tampa Scale for Kinesiophobia; PSEQ: Pain Self-Efficacy Questionnaire; CSI: Central Sensitization Inventory; MCID, minimal clinically important difference; MCD, minimum detectable change; MDC, minimum detectable change

Performance Metric Attributes	Performance Metrics	Units/Score Range	Baseline	Week 8	Score Change	MCID/MCD	MCID/MCD Met?
COPM	Performance (points)	1-10 points	1.7	2.3	0.6	MCID: 2.0 points	No
	Satisfaction (points)	1-10 points	1.0	1.3	0.3	MCID: 2.0 points	No
ROM	Wrist Palmar Flexion (°)	Degrees (°)	50	65	15	MCD: 5°-6°	Yes
	Wrist Dorsal Flexion (°)	Degrees (°)	35	50	15	MCD: 5°-6°	Yes
DASH	Function (points)	0-100 points	78	57	-21	MCID: 10.8 points	Yes
NRS	Pain at Rest	0-10 points	10	3	-7	MCID: 2.0 points	Yes
	Pain During Movement	0-10 points	10	7	-3	MCID: 2.0 points	Yes
PCS	Total (points)	0-52 points	35	25	-9	MCID: 6.0 points	Yes
TSK	Total (points)	17-68 points	48	41	-7	MCID: 4.0-6.0 points	Yes
PSEQ	Total (points)	0-60 points	23	26	3	MCID: 5.0-8.0 points	No
CSI	Total (points)	0-36 points	18	14	-4	MCID: 5.0-8.0 points	No

Therapeutic intervention

The core strategy of the current intervention was to use objective data obtained from a wearable device (Fitbit) to visualize the discrepancy between the patient’s “subjective perception of activity” and “objective physical load.” Specifically, the patient's daily “Active Minutes” (continuous physical activity duration calculated by the Fitbit’s built-in triaxial accelerometer based on metabolic equivalents (METs)) was utilized as the primary metric representing this physical load, rather than simple step counts. Using this visualization as a mirror, the goal was to enable the patient to self-regulate her distorted body perception based on actual physical responses, thereby enhancing her ability to self-regulate and manage pain independently. The appearance of both hands and the placement of the Fitbit are shown in Figure 2. The intervention set “relearning how to use chopsticks” and “resuming occupational writing” - which the patient prioritized - as its ultimate goals and was implemented in the following three phases in accordance with the progression of the condition.

**Figure 2:**
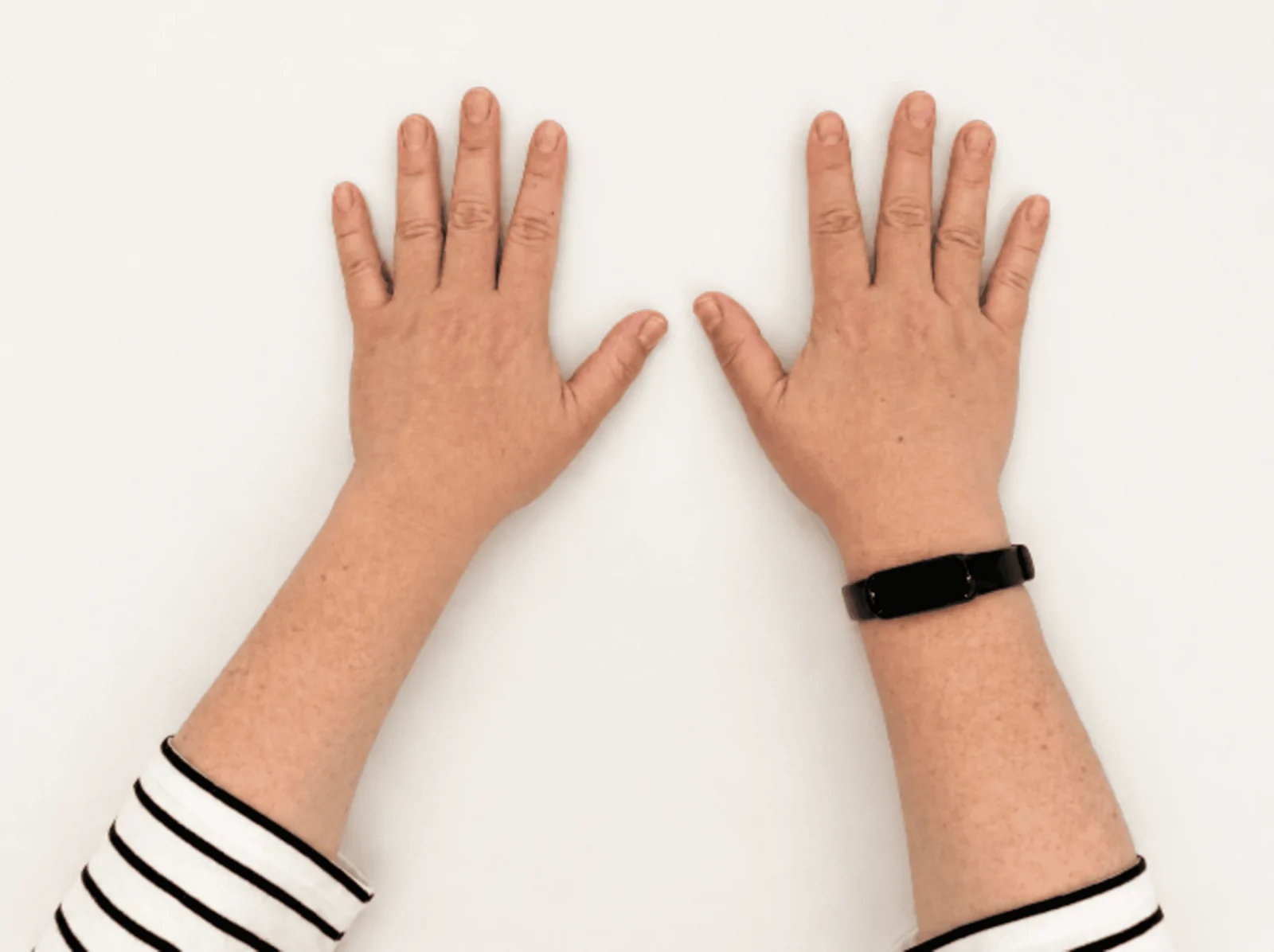
Clinical Appearance of the Patient’s Hands and the Placement of the Wearable Activity Monitor A Fitbit device was worn on the right wrist to continuously track objective activity levels in daily life, serving as a “mirror” for the self-regulation intervention.

The initial phase of the intervention focused on the self-regulation of subjective activity perception based on objective data and the suppression of hyperactivity. The objective of this phase was to improve the “boom-and-bust cycle” - a pattern of alternating disorderly hyperactivity and hypoactivity - by correcting perceptual discrepancies. To achieve this, visualization of activity levels and a three-step feedback process were implemented using a Fitbit to continuously track activity levels during daily life once or twice a week. First, we visually presented instances of unusually high activity levels on specific days and extreme fluctuations in which activity levels plummeted. Subsequently, we shared the discrepancy (recall bias) between the patient’s recollection of “not using it that much” and the actual data showing excessive activity, helping her objectively understand the activity threshold that triggers pain exacerbation. Based on these findings, activity was redefined not as a mere “restriction” but as an “adaptive strategy” to stabilize symptoms and optimize function. To further promote self-regulatory behavior, based on the visualized data, the patient was encouraged to propose and implement specific compensatory measures and environmental adjustments - such as “limiting mopping to five minutes and using the left hand for the rest” or “asking her husband to handle heavy laundry” - thereby successfully reducing the overload on the affected limb.

During the mid-term phase, the focus shifted to modifying “pain predictions” through the reduction of exercise phobia and sensory re-education. The objective of this phase was to correct the discrepancy between the subjective prediction that “contact equals severe pain” and the actual sensory input - and thereby reduce fear of movement - once resting pain had been alleviated through the establishment of self-regulatory behaviors. The intervention was implemented as a desensitization protocol based on strict phasing. First, gradation of stimulus materials was performed by beginning with low-stimulus tissue paper and gradually switching to smooth linen fabric and then to cotton towels with high frictional resistance, depending on the patient’s tolerance. Next, the order of contact sites was carefully managed: we began with the ulnar aspect of the dorsum of the hand, where the response was stable, and gradually expanded the range of contact sites to the radial side (the interdigital space between the thumb and index finger and the dorsal aspect of the proximal phalanx), where allodynia was prominent. Furthermore, we implemented a gradual progression of contact styles, moving from light, point-like contact to surface contact (stroking) and then to contact involving the mild pressure necessary for holding objects. Finally, in each session, the process was repeated until the patient revised her subjective expectation to “with some adaptation, it is okay to be touched” and accumulated a series of gradual successes.

In the final phase of the intervention, the focus was placed on gradual functional loading based on morphological characteristics and integration into daily life. The objective of this phase was to gradually introduce the fine motor skills needed for real-life activities once the fear of touch had been reduced and to integrate these skills into role performance. Specifically, staging based on object characteristics was implemented through grasping training that accounted for the shape, friction, and size of the objects. For instance, we began with beanbags the size of an egg, which fit easily in the palm, and progressed to cube-shaped building blocks and then to spheres, which were more difficult to stabilize. Furthermore, for friction, we shifted from rubber materials that are easy to hold to low-friction, slippery balls, encouraging fine-tuning of the grasping force. Regarding size, we gradually reduced the size of objects to fine items such as pencils, coins, marbles, and nails, thereby demanding greater dexterity. Additionally, strategic selection and focus on activities were carried out in parallel with functional training, and the patient continued strategic self-regulation, deciding “which activities to allocate energy to” while monitoring Fitbit data. Consequently, this approach curbed haphazard energy expenditure and enabled the transition from self-help chopsticks to standard chopsticks, as well as the resumption of interpreting work under restricted conditions.

Progress

During the initial phase of the intervention, the focus was placed on behavioral change through the visualization of overactivity and the correction of the discrepancy between subjective and objective perceptions. At the start of the intervention, the patient experienced severe pain in the right hand (NRS score: 10), and both the performance level and satisfaction with chopstick use were rated at 1 point, indicating a significant decline. Concurrently, the patient held strong fixations regarding dining etiquette and the belief that “food doesn’t taste good unless eaten with my dominant hand.” Consequently, she continued to use her right hand despite pain, and she repeatedly fell into maladaptive activity patterns (boom-and-bust cycles) that left her bedridden for several days.

The Fitbit baseline data revealed a marked imbalance between periods of excessive activity and inactivity (Figures 3A-3B). Specifically, her daily active minutes spiked to 52 minutes on POD 78, when she completed both rehabilitation and driving, which was immediately followed by a complete crash to 0 minutes on POD 82 due to severe pain and headache (Figure 3B). However, following the pacing intervention, her daily active minutes stabilized during the final phase (all remaining below 45 minutes per day from POD 113 to 123), demonstrating the transition to consistent Activity Pacing.

**Figure 3:**
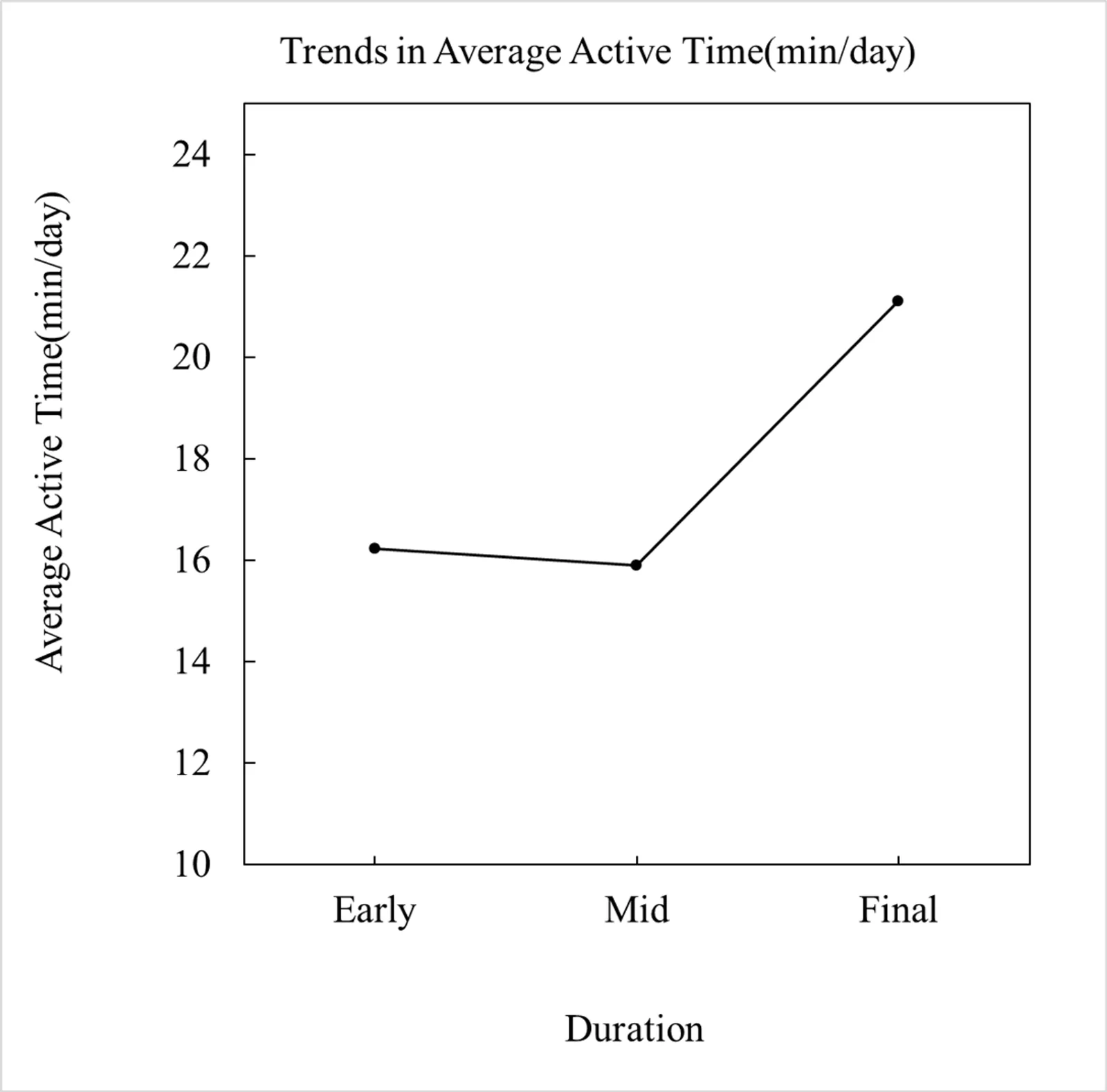
Upper-Limb Active Time Trends Longitudinal trend showing the average active minutes per day across the early, mid, and final phases of the intervention.

When the occupational therapist presented this objective activity data, the patient expressed surprise at the discrepancy between her subjective perception of “not using it that much” and her actual activity levels. Through this discrepancy, the patient became aware of her recall bias, which served as a catalyst for her to objectively recognize the activity threshold that triggers pain exacerbation. Consequently, starting in the second week of intervention, the patient began to independently propose compensatory strategies, such as “consciously mopping with my left hand” and “avoiding heavy laundry.” The participant also remarked, “I realized that consciously limiting my activity reduced the tightness in my hand.” Based on these developments, it is possible that feedback on objective activity levels helped the patient visualize the discrepancy in her perception, further encouraging her to self-regulate her activities.

During the mid-term phase, the pacing (self-regulation) of activities acquired in the initial phase led to a reduction in pain at rest. Subsequently, an intervention was implemented with the aim of correcting the distorted body perception that “contact = severe pain.” In order to prevent learned non-use of the right upper limb and facilitate functional grasping, a desensitization protocol was implemented in which the texture of the stimulus material (tissue → linen → cotton towel) and the contact site (ulnar side of the back of the hand → radial side) were gradually adjusted. By repeating this gradual sensory input, the patient eventually began to state, “If I’m careful, it’s okay to be touched.” This change may have contributed to a reduction in excessive fear of contact and avoidance behaviors; furthermore, it may have served as a supporting factor for subsequent high-intensity functional training and the patient’s transition to ADL.

In the final phase, the patient’s wariness about physical contact had diminished by the end of the intervention period. Consequently, the workload for fine motor tasks was gradually increased based on the morphological characteristics of the objects (shape, friction, and size). To achieve the ultimate goals of regaining standard chopstick use and occupational writing skills, the tasks were progressively shifted from beanbags to building blocks and then to fine objects such as nails and coins. Throughout the intervention period, objective activity levels were continuously monitored using a Fitbit, with the occupational therapist providing continuous feedback on the patient's results. As a result, the patient began adjusting her ADL based on objective activity levels and, additionally, began exhibiting behaviors that reflected a conscious effort to “prioritize energy allocation for specific activities.” At the eight-week mark of the intervention, the patient remarked, “If I try hard, I can grip with my index finger,” which suggests an improvement in self-efficacy. In the final evaluation, the DASH score improved by 21 points, from 78 to 57, and this improvement exceeded the minimum clinically important difference (MCID) of 10.8 points. Furthermore, the patient resumed her interpretation work with a limited writing workload, confirming that a concrete recovery of role performance was observed. Based on these findings, feedback on objective activity levels may have contributed to the patient's recognition of the discrepancy between her subjective perception of activity and her actual objective activity levels.

Longitudinal trend showing the average active minutes per day across the early, mid, and final phases of the intervention. Representative daily active minutes and their trends generated from the exported Fitbit data during the initial phase (POD 78 to 89, showing the “boom-and-bust” fluctuation from 52 minutes on POD 78 to 0 minutes on POD 82) and the final phase (POD 113 to 123, showing stabilized active minutes of under 45 minutes/day) (Figure 4).

**Figure 4:**
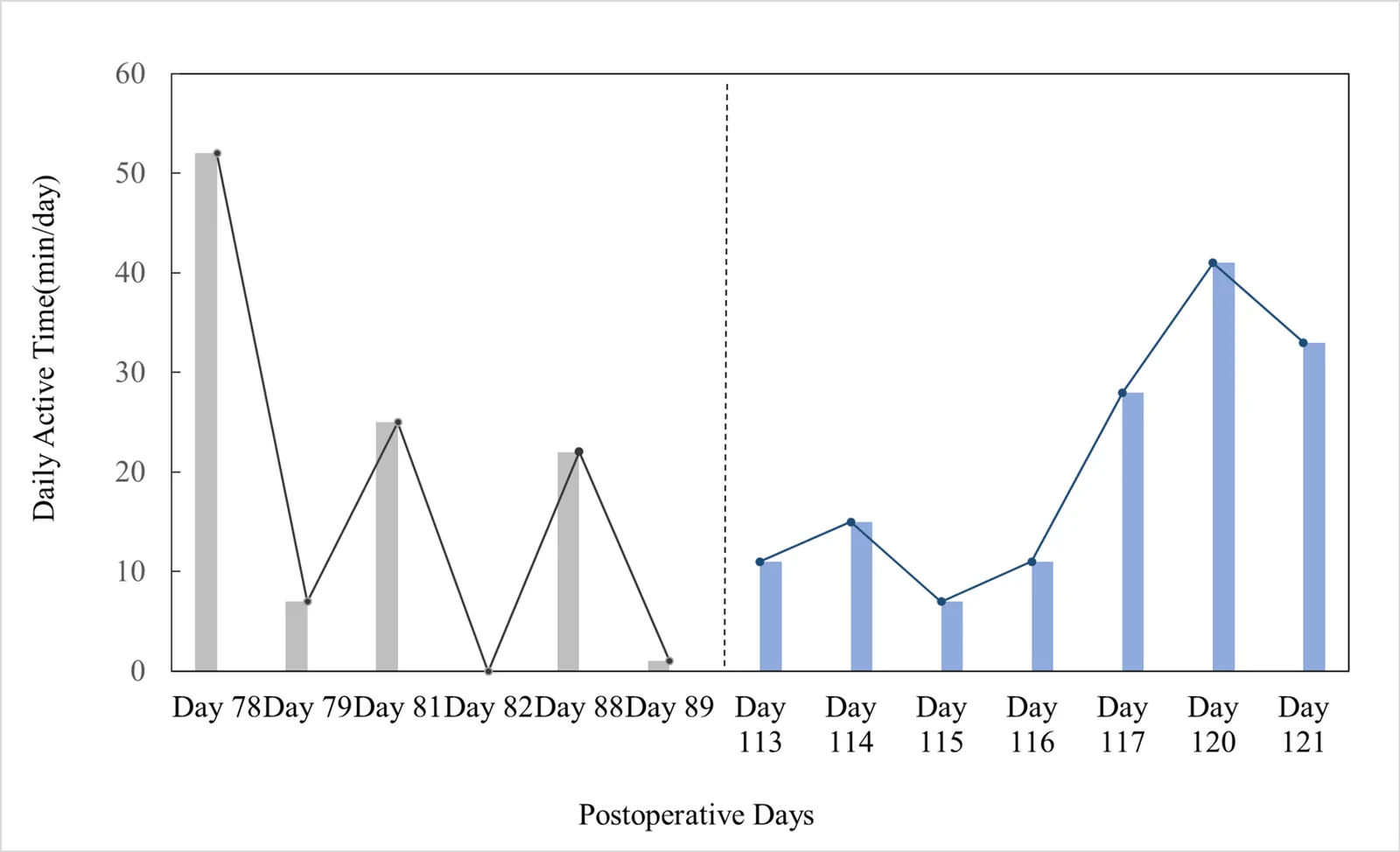
Daily Active Minutes and Pacing Trends Representative daily active minutes and their trends generated from the exported Fitbit data during the initial phase and the final phase.

Results

Table 1 presents the assessment results conducted eight weeks after the initial assessment, reflecting the changes before and after the intervention. The upper limb functional impairment score, assessed using the DASH score, improved from 78 to 57; this 21-point improvement exceeded the MCID of 10.8 points. Additionally, catastrophic thinking regarding pain, assessed using the PCS, improved from 35 to 26 points, a nine-point gain that exceeded the MCID of 6 points and brought the score below the cutoff value of 30. Furthermore, the central sensitization index, assessed using the CSI-9, decreased from 18 to 14, whereas NRS scores showed a marked reduction from the baseline of 10 to a resting pain score of 3 and an activity-related pain score of 7 at the final assessment. Although there was some fluctuation in pain levels, a reduction was observed compared with pre-intervention levels, and performance on chopstick use - the primary goal identified in the COPM - improved from 1 to 3 points, as shown in the practice session illustrated in Figure 5.

**Figure 5:**
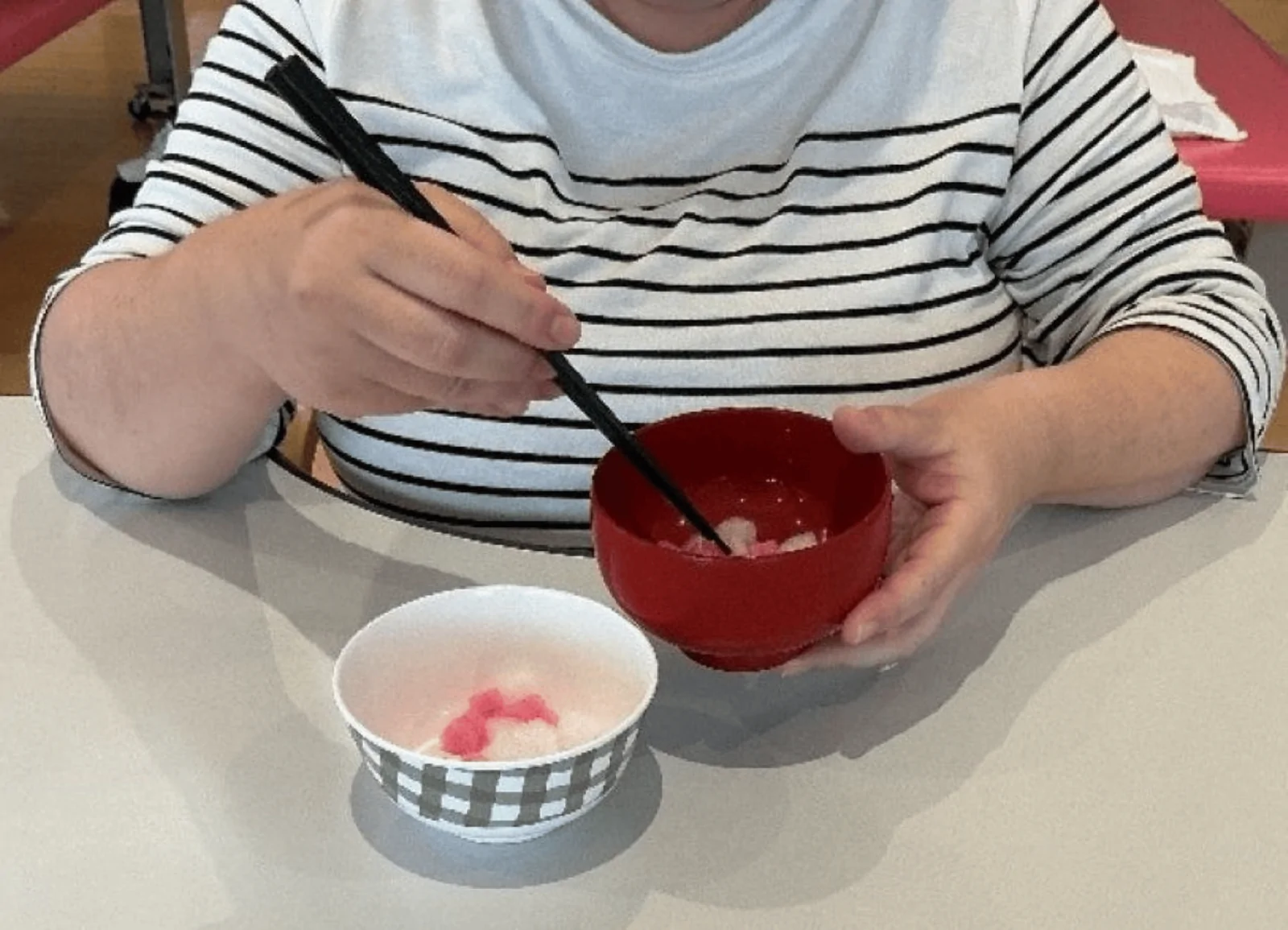
Occupational Therapy Session Focusing on the Re-acquisition of Functional Chopstick Use Practical training was conducted to achieve the patient’s primary goal of "being able to eat using chopsticks," which was identified as a high-priority task during the initial COPM assessment. COPM: Canadian Occupational Performance Measure

However, satisfaction remained at 1 point, and the change in the average COPM performance score was 0.6 points, which did not reach the MCID of 2.0 points. Similarly, fear of movement, assessed using the TSK, improved from 48 to 41 points, but the score still exceeded the cutoff value of 37 points. Moreover, self-efficacy, as assessed using the PSEQ, increased from 23 to 26, though this change was small and did not reach the cutoff value of 40 points. Despite the fact that some psychological indicators and COPM satisfaction scores did not reach the reference values, the patient acquired the ability to use the Fitbit data as a “mirror for self-regulation.” Rather than fixating on the pain score itself, the patient began adjusting activities with a conscious focus on “which tasks to allocate energy to”; consequently, adaptive self-management behaviors based on “selection and concentration of activities” became established.

## Discussion

In this study, we implemented an intervention that combined objective activity feedback via a wearable device with goal-based Activity Pacing. Consequently, the “boom-and-bust” cycle, characterized by alternating periods of overactivity and inactivity, seemed to have improved. Furthermore, improvements in upper limb function and certain aspects of task performance were observed [5,6]. Additionally, the DASH score improved from 78 to 57, representing an improvement of 21 points and exceeding the MCID of 10.8 points [15]. The PCS score improved from 35 to 26, representing a 9-point improvement and exceeding the MCID of 6 points. Additionally, the PCS score fell below the cutoff value of 30 points [12]. However, improvements in the PSEQ and COPM satisfaction scores were limited. These results suggest that Activity Pacing, which incorporates feedback on objective activity levels, may support the self-regulation of activity and contribute to improvements in functional impairment and pain perception.

In this case, the patient was engaged in the professional field of interpretation and translation, which required a high degree of accuracy and a strong sense of responsibility. Additionally, the patient held a strong sense of role expectation, believing that “I must perform household chores perfectly.” This may have contributed to overactivity, in which the patient continued her activities despite experiencing severe pain. Furthermore, distorted body perception of the affected limb has been reported in patients with early-stage CRPS, which is an important target for intervention [16]. Such distorted body perception may make it difficult to accurately self-assess activity levels and may contribute to a “boom-and-bust” cycle of alternating overactivity and inactivity.

Indeed, the patient’s baseline resting and movement pain scores of 10/10 were consistent with severe hypersensitivity and may have reflected disturbances in body perception and central sensitization. Under these conditions, altered central pain processing may generate heightened pain responses that are disproportionate to actual peripheral loading, thereby blurring the subjective distinction between rest and active movement. Early Fitbit data from the intervention period recorded a notable 52-minute stretch of continuous activity on a specific day, with activity levels dropping sharply to 0 min a few days later. Such extreme fluctuations in activity levels suggest that the patient may not have had a sufficient subjective understanding of the activity threshold that triggers pain exacerbation.

Nielson et al. defined “Activity Pacing” not merely as activity restriction but as an adaptive strategy to minimize symptom fluctuations and optimize function [17]. In this case, receiving feedback on objective activity data may have provided the patient with an opportunity to recognize the activity levels likely to trigger pain exacerbation. Subsequently, she began to independently select specific compensatory measures, such as “switching the mop to the left hand” and “avoiding heavy laundry.” These changes indicate that the patient, while recognizing the discrepancy between her subjective perception of activity and objective activity levels, transitioned to behavior involving self-regulation of activity. This process may have been associated with a transition from the motivational stage to the volitional stage in the Health Action Process Approach theory [18].

Our results suggest that Activity Pacing, which incorporates feedback on objective activity levels, may support the self-regulation of activity, contribute to improvements in functional impairment and pain perception, and reduce overload on the affected limb. Furthermore, this self-regulation may have served as the foundation for subsequent phased sensory re-education and functional training. Specifically, this severe body perception distortion was addressed by the graded desensitization protocol (tissue → linen → cotton) in Phase 2. This systematic tactile progression provided predictable, non-threatening sensory inputs to the hypersensitive limb, aiming to correct the brain’s exaggerated pain predictions, restore normal cortical representation, and reduce touch-related kinesiophobia.

The results of this case are generally consistent with those of previous studies that focused on the discrepancy between the subjective perception of activity and objective activity levels. Tynan et al. reported that self-reported and objective activity levels measured using sensors did not align in patients with chronic pain [5]. This highlights the importance of objective assessments that are less susceptible to recall bias. While scientific-grade accelerometers often require specialized software for retrospective analysis, consumer-grade activity trackers like Fitbit provide patients with immediate, understandable visual feedback. This clinical accessibility not only minimizes user burden but also enhances the feasibility of incorporating wearable device-based feedback into routine, real-world occupational therapy settings. In this case, a discrepancy was also observed between the patient’s subjective perception of “not using the operated limb” and the objective activity level recorded by the Fitbit. Feedback on objective activity levels may have served as a catalyst for the patient to recognize this discrepancy. Avila et al. reported that activity feedback from wearable devices could serve as an intervention tool to support self-regulation [19]. In this case, it is also possible that, through feedback on objective activity levels, the patient became aware of the discrepancy between her subjective perception of activity and her actual objective activity levels. Subsequently, the patient accumulated successful experiences, such as “I can avoid worsening pain by spreading out my activities” and “I can grip with my index finger if I use a different technique.” Furthermore, the patient began to practice self-regulation of activities, such as “switching the mop to my left hand” and “avoiding heavy laundry.” These changes are consistent with Avila et al.’s report [19] that feedback on objective activity levels supports self-adjustment. In our first study, we reported that pain intensity six months after surgery for LE was more strongly associated with PREE-J, a patient-reported measure of subjective functional impairment, than with objective indicators of physical function, such as grip strength and range of motion [8]. This finding highlights the importance of assessing not only physical function but also the patient’s subjective perception of impairment. We used feedback on objective activity levels to visualize the discrepancy between the patient’s subjective perception of activity and her actual objective activity levels. Consequently, patients may begin to self-regulate their activities. The results of this case suggest that feedback on objective activity levels may function as an intervention to identify the discrepancy between subjective perception and objective activity levels and link this discrepancy to self-regulation.

As this is a single-case study, there are limitations to generalizing the results to all patients with CRPS. Furthermore, because the program was multifaceted - concurrently incorporating activity feedback, pacing, sensory desensitization, and gradual loading - it was impossible to isolate the independent therapeutic effect of the Fitbit feedback alone. Nevertheless, functional impairment (DASH score) and pain perception (PCS) improved beyond the MCID. However, satisfaction with chopstick use remained low. Moreover, as a single-case report, these findings must be interpreted strictly as preliminary observations and clinical hypotheses rather than definitive evidence of therapeutic efficacy. Prospective, larger-scale controlled trials are strictly required to objectively verify and establish the actual clinical efficacy of this approach. These results may have been influenced by the patient’s perfectionist personality traits and self-evaluation standards that demanded a high level of performance as a professional. Furthermore, these results suggest that, even when self-regulation of activity is encouraged through feedback on objective activity levels, such changes do not necessarily lead directly to improved subjective satisfaction. Hence, future large-scale intervention studies may be needed to validate our findings in the general patient population. Moreover, prospective studies verifying how this approach contributes to visualizing the discrepancy between the subjective perception of activity and objective activity levels, as well as to the self-regulation of activity, are warranted.

## Conclusions

In this case report, we implemented a multifaceted occupational therapy program that integrated feedback on objective activity levels from an activity monitor with goal-based Activity Pacing for a patient with early-stage CRPS exhibiting a “boom-and-bust” cycle. Consequently, disorganized hyperactivity seemed to improve in this case. Furthermore, catastrophic thinking regarding pain and upper limb functional impairment also improved. By visualizing objective activity levels, which may have helped the patient recognize the discrepancy between her subjective perception of activity and her actual objective activity levels, the patient may have been enabled to self-regulate her activities. Although this approach presents a plausible clinical hypothesis with the potential to serve as an occupational therapy strategy to support self-management in patients with chronic pain, including those with early-stage CRPS, these preliminary observations do not constitute direct evidence of therapeutic efficacy. Therefore, prospective, larger-scale controlled studies are strictly required to verify and establish the clinical effectiveness of this intervention.
